# The use of surgical rating scales for the evaluation of surgical working conditions during laparoscopic surgery: a scoping review

**DOI:** 10.1007/s00464-018-6424-5

**Published:** 2018-09-14

**Authors:** Martijn Boon, Christian H. Martini, Leon P. H. J. Aarts, Albert Dahan

**Affiliations:** Department of Anesthesiology, Leiden University Medical Center, Albinusdreef 2, 2333 Leiden, The Netherlands

**Keywords:** Laparoscopic surgery, Intraoperative conditions, Surgical rating scale

## Abstract

**Introduction:**

Surgical rating scales (SRSs) enable the surgeon to uniformly quantify surgical working conditions. They are increasingly used as a primary outcome in studies evaluating the effect of anaesthesia or surgery-related interventions on the quality of the surgical work field. SRSs are especially used in laparoscopic surgery due to a renewed interest in deep neuromuscular block. There are however no guidelines regarding the uniform use of SRS and the uniform reporting of results.

**Methods:**

A systematic search was conducted in the databases of PubMed, Web of Science and Embase for studies that reported the use of an SRS to evaluate surgical conditions in laparoscopic surgery. Only original human research in English language with full text availability through the Leiden university library was considered for this review. The full texts of eligible abstracts were independently reviewed by the first and second author. The quality of SRSs and methodology of rating were systematically reviewed.

**Results:**

The search yielded 2830 reports, of which 17 were identified using a surgical rating scale (SRS) in laparoscopic surgery. Ten of these reports used a unique SRS, these were systematically appraised for their quality. The overall quality of the SRSs was low: the majority of the scales were poorly described and lacked assessment of inter- and intra-rater reliability. In addition, considerable differences exist in the methodology of rating and the reporting of results.

**Conclusion:**

There is substantial inconsistency in SRS quality, methodology, and results reporting. The uniform use of high-quality surgical rating scales is needed to improve the quality and reproducibility of future research.

Surgical rating scales (SRS) are increasingly used to rate the quality of surgical working conditions. A SRS enables the surgeon to translate his or her experienced but subjective impression of the quality of the operative conditions into a standardised rating. The use of SRSs has potential benefits in daily practice and research. First, it offers a uniform platform for the surgeon to negotiate with the anaesthetist whether or not to improve or consolidate surgical working conditions induced by the anaesthetic. Second, surgical rating scales may be used in research to evaluate interventions and new techniques aimed at improving the surgical working/operating conditions. Recent developments in the reversal of neuromuscular block by sugammadex have renewed the interest in the effect of deeper levels of neuromuscular block on surgical working conditions in laparoscopic surgery. In these studies, surgical rating scales are often used as primary outcome [1–8]. However, guidelines on the use of surgical rating scales do not exist as yet. This systematic review gives an overview of the use of SRSs in laparoscopic surgery and proposes guidance for future research.

## Methods

The first author conducted a literature search assisted by the librarian of the Leiden University Medical Centre. The following query was used to search the pubmed database: (“rating scale” [tw] OR “rating scales” [tw] OR “Visual Analog Scale” [Mesh] OR Visual Analogue Scale* [tw] OR Visual Analog Scale* [tw] OR “scale” [tw] OR “scales” [tw] OR scaling* [tw] OR rating* [tw] OR scoring* [tw] OR “score” [tw] OR “scores” [tw] OR “scored” [tw] OR “grading” [tw] OR “grade” [tw] OR “graded” [tw]) AND (“surgical conditions” [tw] OR “surgical condition” [tw] OR “operating conditions” [tw] OR “operating condition” [tw] OR “surgical quality” [tw] OR “surgery quality” [tw] OR “surgical field” [tw]). Embase and Web of Science were searched with a similar query containing the following terms: “rating scale”, “visual analogue scale” (included Mesh term), “scale”, “rating”, “scoring”, “score”, “grading”, “surgical conditions”, “operating conditions”, “surgical quality”, and “surgical field”. The databases were searched on the 20th may 2017, without date range limit. The results were screened on title and abstract by the first author. Relevant full text articles were retrieved and the reference lists of these articles were screened for any additional missed papers (snow ball method). After this first selection, the full texts of the selected articles were reviewed by the first and second author for inclusion in the review.

### Study inclusion criteria

Studies included in this systematic review were limited to original randomised controlled trials, English language and full text availability through the Leiden University full text access service. Articles were included if the study (1) described a method to evaluate a surgical working condition or operating field or (2) applied a surgical rating scale for evaluation of surgical conditions in laparoscopic surgery. Included publications were assessed for the following items: type of rating scale, description of the scale items, number of raters, scoring moments, validation methods, and reporting of results.

### Exclusion criteria

Reports that did not score surgical conditions as a whole, but only specific subparts such as “satisfaction of the surgeon”, were excluded.

### Quality assessment of the rating scales

In general, the quality of a measurement instrument is critically dependent on its construct validity and reliability [9, 10]. Construct validity refers to the quality of the data based on the scores from a measurement instrument and whether it adequately represents the underlying construct (i.e. the surgical working conditions) [9]. For construct validity, the following domains are considered important: scale content, internal structure, response process, correlation to other variables, and clinical consequences [9–11]. These domains reflect both the internal quality of the rating instrument (scale content, internal structure, correlation to other variables) and how the rating instrument is used in practice (scoring methodology; response/rating process). To uniformly assess the quality of the identified SRSs in this review, an appraisal score was constructed. We are not aware of any pre-existing scores for the appraisal of surgical rating scales. In the appraisal score, relevant previously mentioned domains were translated into the following psychometric items: (1) scale length, (2) description of the scale items, (3) test–retest reliability, and (4) correlation with other variables (see Table 1). The appraisal score only assesses internal SRS quality; the scoring methodology is discussed separately. All SRSs were independently reviewed by the first and second author with the use of the appraisal score. Discrepancies were resolved by consensus. We will briefly explain the separate items of the appraisal score.

**Table 1:** Appraisal score used to grade the surgical rating scales

Length of the scale	Points
< 5 items	0
5–7 items	1
> 7 items	0
Scale Item definition	
Inadequate	0
Adequate	1
Reliability assessment	
None	0
Inter-rater reliability	1
Intra-rater reliability	1
Both	2
Correlation with other variables	
No	0
Yes	1

#### Length of the SRS

An SRS length of 5–7 items is considered optimal. Test–retest reliability, internal consistency, and discriminating power of scales with 5–7 items are generally superior to short scales (2–4 item points) or very large scales (> 10 item points) [12, 13]. In our appraisal score, scales with a length of 5–7 items received one point. Scales that contained less than 5 or more than 7 items were not granted any points.

#### Description of scale items

In a well-described scale, *each* item in the scale has a grade (i.e. moderate or excellent) *plus* a detailed description of the specific aspects of the surgical working field for that grade. An example of an SRS with an adequate scale item description is the Leiden-surgical rating scale. This scale is presented in Table 2 [1]. Scales that have an adequate description of the scale items were granted one point in the appraisal score. Inadequate or absence of detailed description of the scale items resulted in zero points in the appraisal score.

**Table 2:** Leiden-surgical rating scale [1]

1	*Extremely poor conditions* the surgeon is unable to work because of coughing or because of the inability to obtain a visible laparoscopic field because of inadequate muscle relaxation. Additional neuromuscular blocking agents must be given
2	*Poor conditions* there is a visible laparoscopic field, but the surgeon is severely hampered by inadequate muscle relaxation with continuous muscle contractions, movements, or both with the hazard of tissue damage. Additional neuromuscular blocking agents must be given
3	*Acceptable conditions* there is a wide visible laparoscopic field but muscle contractions, movements, or both occur regularly causing some interference with the surgeon’s work. There is the need for additional neuromuscular blocking agents to prevent deterioration
4	*Good conditions* there is a wide laparoscopic working field with sporadic muscle contractions, movements, or both. There is no immediate need for additional neuromuscular blocking agents unless there is the fear for deterioration
5	*Optimal conditions* there is a wide visible laparoscopic working field without any movement or contractions. There is no need for additional neuromuscular blocking agents

#### Test–retest reliability

Test–retest reliability assesses the reproducibility of ratings by one rater (intra-observer reliability) or between two (or more) raters (inter-observer reliability). At best, an SRS was assessed for both. The appraisal score grants one point for intra-observer and one point for and inter-observer reliability verification. Hence, the maximum score in the appraisal score for this domain was two points.

#### Correlation with other variables

According to the domains of construct validity, a measurement instrument should be compared to another measurement instrument or variable that reflects the same underlying construct. In the appraisal score, if an SRS was compared with another scoring instrument or variable, it received one point. The absence of such a comparison would result in zero points.

The appraisal scoring system is given in Table 1. The maximum score that an SRS could receive was five points (excellent quality) and the lowest score was zero points (very poor quality).

## Results

### Included articles

**Fig. 1:**
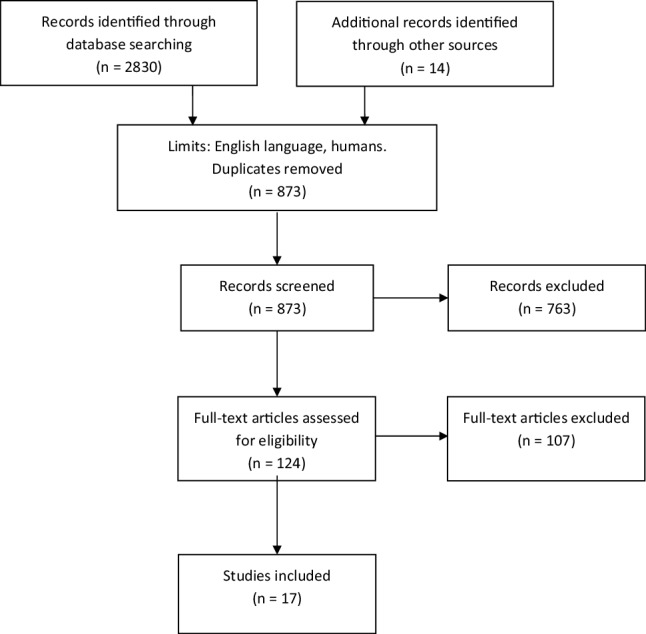
Study flow-chart

The initial search yielded 2830 publications. After removing duplicates, non-English language and non-human research, we screened 873 abstracts of which 763 non-relevant publications were discarded. The full texts of 110 reports were reviewed. The snowball method yielded 14 additional relevant studies. After full text review of 124 selected articles, 15 reports were excluded because (1) the SRS was not used for assessment of surgical conditions, or (2) surgical conditions were not scored. Another 92 reports were excluded because of non-laparoscopic surgery (3). In total, 17 publications were included in this review. Figure 1 outlines the selection process. The unique SRSs were systematically judged for their quality with the use of the appraisal score. Overall, the quality of the majority of the SRSs was low (see Table 3).

**Table 3:** Quality score per surgical rating scale

Author	Year	Specialty	Scale length	Item description	Reliability assessment	Correlation with other variables	Total
Martini [1]	2013	Urology	1	1	2	1	5
Caldwell [14]	1985	Gynaecology	0	1	0	0	1
Madsen [15]	2015	Gynaecology	1	0	0	1	2
Williams [16]	2003	Gynaecology	1	0	0	1	2
Dubois [2]	2014	Gynaecology	0	0	0	0	0
Blobner [3]	2014	General Surgery	0	0	0	1	1
Koo [17]	2016	General Surgery	1	0	0	1	2
Kim [6]	2016	General Surgery	1	0	0	0	1
Rosenberg [18]	2017	General Surgery	0	0	0	1	1
Taylor [19]	1992	General Surgery	1	0	0	1	2

0 = very poor quality; 5 = excellent quality

### Surgical rating scales used in laparoscopic surgery

Seventeen studies used a SRS for evaluation of surgical conditions in laparoscopic surgery [1–7, 15–24]. The length of the individual scales varied between 3-, 4-, 5-, 6-, 11-, and 100-point scales. Most surgical rating scales were 4- or 5-point scales (see Table 4).

**Table 4:** The use of surgical rating scales in laparoscopic surgery

Author	Year	Specialty	Comparison	Scale	Scale description	Raters (*n*)	Interval	Outcome
Martini [1]	2014	Urology	Deep versus moderate NMB	5 point^a^	1 (extremely poor)–5 (optimal)	1	15 min	Mean SRS, % subopt./opt. cond
Yoo [21]	2015	Urology	Deep versus moderate NMB	5 point^a^	1 (extremely poor)–5 (optimal)	1	End of surgery	Mean SRS
Boon [7]	2016	Urology	Deep versus moderate NMB	5 point^a^	1 (extremely poor)–5 (optimal)	1	15 min	Mean SRS, % subopt./opt. cond
Torensma [4]	2016	Bariatric surgery	Deep versus moderate NMB	5 point^a^	1 (extremely poor)–5 (optimal)	3	10 min	Mean SRS, % subopt./opt. cond
Baete [5]	2017	Bariatric surgery	Deep versus moderate NMB	5 point^a^	1 (extremely poor)–5 (optimal)	1	End of surgery	Mean SRS
Caldwell [24]	1985	Gynaecology	NMB	3 point	1 (good)–3 (inadequate)	Unknown	Unknown	SRS distribution
Williams [16]	2003	Gynaecology	Moderate versus no NMB	4 point	1 (poor)–4 (excellent)	Unknown	Unknown	SRS distribution
Dubois [2]	2014	Gynaecology	Deep versus moderate NMB	4 point	1 (optimal)–4 (unacceptable)	1	10 min	Mean SRS, SRS distribution
Madsen [15]	2015	Gynaecology	Deep versus no NMB	4 point	1 (optimal)–4 (bad)	2	Fascia closure	Mean SRS, intra-abdominal space (cm)
Taylor [19]	1992	General surgery	Nitrous oxide	5 point	1 (extremely poor)–5 (very good)	1	15 min	SRS, bowel distention
Staer Rye [20]	2014	General surgery	Deep versus moderate NMB	4 point	1 (optimal)–4 (unacceptable)	2	Multiple	% subopt./opt.cond, completion IAP 8 mmHg
Blobner [3]	2014	General surgery	Deep versus no NMB	101 point	0 (not acceptable)–100 (excellent)	Unknown	End of surgery	% subopt./opt. cond
Koo [17]	2016	General surgery	Deep versus moderate NMB	4 point	1 (excellent)–4 (poor)	Unknown	End of surgery	% subopt./opt. cond., increase IAP (*n*)
Kim [25]	2016	General surgery	Deep versus moderate NMB	5 point^a^	1 (extremely poor)–5 (optimal)	Unknown	End of surgery	SRS, titrated IAP
Rosenberg [18]	2017	General surgery	Deep versus moderate NMB	11 point	0 (poor)–10 (excellent)	Unknown	End of surgery	Mean SRS, SRS distribution
Ozdemir [22]	2017	General surgery	Deep versus moderate NMB	5 point^a^	1 (extremely poor)–5 (optimal)	Unknown	15 min	Mean SRS, SRS distribution
Ozdemir [23]	2017	General surgery	Deep versus moderate NMB	5 point^a^	1 (extremely poor)–5 (optimal)	Unknown	15 min	Mean SRS, SRS distribution

*SRS* surgical rating scale, *% subopt*./*opt. Cond*. percentage of suboptimal/optimal conditions, *NMB* neuromuscular block, *IAP* intra-abdominal pressure

^a^Leiden-surgical rating scale

Four-point scales are commonly used for evaluation of surgical conditions, predominantly laparoscopic gynaecologic surgery [2, 15, 16, 20]. However, in the quality appraisal, these 4-point scales were rated as poor-quality scales as the length of 4-point scales was considered suboptimal (< 5 items) and all lacked test–retest reliability assessment.

Taylor et al. used a 5-point SRS to assess surgical conditions during cholecystectomy in relation to bowel distension and the use of nitrous oxide [19]. This scale also lacked test–retest reliability assessment. Martini et al. developed their 5-point Leiden-surgical rating scale (L-SRS) for use in laparoscopic retroperitoneal urologic surgery (see Table 2) [1]. The scale was later successfully used in bariatric surgery [4]. The scale items are well described and incorporate visibility of critical structures, working space, and muscle contractions as determinants of the surgical working field [1]. The 5-point L-SRS was assessed for inter-rater reliability by the original research group [1, 4]. In addition, Nervil et al. assessed both inter and intra-rater reliability of a modified version of the 5-point L-SRS and an 11-point SRS [13]. Both the 5-point and 11-point SRS showed excellent intra-rater reliability and fair inter-rater reliability. Due to the lower inter-rater variability, the 5-point scale was considered superior [13]. The L-SRS scale is used by other research groups, including the use in laparoscopic donor nephrectomy [5, 21–23]. This endorses the utility of this scale. In laparoscopic donor nephrectomy, the L-SRS is used to titrate insufflation pressures to the lowest possible, while maintaining good operating conditions.

### Methodology and results reporting

Most studies reported a mean SRS score and a distribution of the scores (see Table 4). Some only reported the percentage of unacceptable surgical conditions, which was generally the frequency of scores on the lower half of the surgical rating scale [3, 17, 20]. In addition, the number and moments of scoring differed considerably, with some studies scoring every 10 or 15 min [1, 2, 4, 7, 19, 20], while others scored one overall score at the end of surgery [3, 5, 8, 15, 17, 18, 21]. Some reports do not mention a scoring interval at all [16, 24]. In addition to the surgical rating scale, some have assessed other outcomes as well such as intra-abdominal space and the effect on insufflation pressures (see Table 4) [6, 15, 17, 20].

## Discussion

Surgical rating scales (SRS) are increasingly used in clinical research. These scales are used to translate the subjective perception of the surgical field by the surgeon into a more objective and reproducible integer on a fixed scale. Surgical rating scales are a useful tool to investigate the effect of surgery- or anaesthesia-related interventions on surgical working conditions. To get an indication on the variety of SRS in use and their quality, we retrieved 17 relevant studies from the literature and identified 10 unique scales that are used in laparoscopic surgery. Since the introduction of sugammadex (a novel selective neuromuscular reversal agent), there has been a renewed interest in the application of deep neuromuscular block (NMB) in these types of surgery. This type of research relies heavily on the use of a SRS.

Based on our results, it is evident that the large number of rating scales in literature comes with significant heterogeneity. There is ample difference in the quality of the rating scales and second, there is no uniformity in the method of rating and reporting of the results. In general, the quality of the rating scales was low. Most encountered problems were absence of test–retest reliability assessment, absence of a comparison with a different scoring instrument, and poor definition of the scale items. Only the Leiden-surgical rating scale received the highest quality score (see Table 3).

The methods of rating (rating methodology) and the reporting of the results of each study were also reviewed and revealed significant differences (see Table 4). For example, the moment of rating (at fixed time points versus at the end of surgery) and the number or raters (one vs. multiple) differed per study or was not detailed in the “Methods” section. This methodologic heterogeneity may impact results considerably. For instance, a surgical rating that is obtained at fixed time points during a procedure, i.e. every 15 min, may give a completely different result compared to one “overall rating” rating at the end of a procedure [4, 5]. Furthermore, the reporting of the SRS results varied considerably, with some reporting means or medians of the SRS, and others only the distribution of the SRS.

In this review, we aimed to uniformly appraise the quality of the identified SRS. To be useful instruments, SRS should display good psychometric properties, such as reliability and validity, and be also easy to use [9–11]. To this end, we created an appraisal score that was used to review these aspects of each SRS (see Table 1). The appraisal score allowed us to uniformly assess the quality of each SRS. Note, however, that the appraisal score is not evidence for validity of the results obtained with the SRS. Both validity and reliability are not inherent properties of the rating instrument, but they rather reflect the interaction of the scale with the measure being tested. We are aware that our appraisal score may possess shortcomings and lacks formal validation. Therefore, others may judge the quality of the SRS differently. Finally, it is important to realise that only English language literature was searched and that useful, high-quality rating scales may exist in non-English literature. In addition, high-quality surgical rating scales may exist in non-laparoscopic surgery, however, this is beyond the scope of this review.

The use of poor-quality SRS combined with poor rating methodology for research is undesirable, and reduces the validity of the results. While we do not intent to recommend a preferred SRS for specific procedures, we do propose some guidance in the use of SRSs. If a good-quality SRS in the field of interest is available, researchers should strongly consider using that scale. The use of existing SRSs increases the comparability of research. If validated SRSs are unavailable for specific surgical procedures, investigators can either choose to validate a pre-existing non-validated scale, or develop and validate a new scale. Any new developed scale should be of high quality. The items mentioned in the appraisal score can act as a guideline for this. The validation procedure should assess both inter- and intra-rater reliability of a scale. In addition, the scale should be compared with other variables to increase its validity. See Table 5 for an overview of recommendations.

**Table 5:** Guideline for future research

1. Surgical rating scale
–Researches should only use pre-existing, validated scales available in their field of research, or (if unavailable)
–Validate a pre-existing, high-quality, non-validated scale in the field of interest (i.e. assessment of inter- and intra-observer reliability), or
–Develop and validate a new surgical rating scale with respect to the domains in the appraisal score
2. Use of the rating scale
–Rating at multiple predefined moments during a procedure (instead of one rating at the end)
–Report number and experience of scoring surgeon raters
3. Reporting of results
–Mean and/or median overall score
–Mean/median score at every rating moment during a procedure
–Distribution of the scores
–Clearly define (un)acceptable conditions (if applicable)
–Compare SRS with other important variables

Finally, ratings should be obtained at predefined moments and researches should report the following in their methods and results: number of individuals involved in the scoring and their surgical experience, time-stamp of scoring, mean and/or median SRS values, mean/median scorings at each time-stamp, and the distribution of the scorings. Uniformity of these aspects will improve comparability and reproducibility of this type of research.

In conclusion, this review found that multiple surgical rating scales have been used in laparoscopic surgery to assess the quality of the surgical field. The majority of the scales are of low quality and the method of rating and reporting of results differed considerably. The uniform use of high-quality surgical rating scales is needed to improve the quality and reproducibility of future research.
